# A Case Report of Growth Hormone–Secreting Pituitary Adenoma Complicated by Apoplexy With Atypical Clinical Presentation

**DOI:** 10.1155/carm/4124145

**Published:** 2026-01-30

**Authors:** Bassem Al Hariri, Muhammad Faizan, Reynaldo Balintona, Mohammed Omer Elbadawi Elhassan, Suhib Salameh, Imran Hussain Mohammad, Abdulwahab Muhammad Qasem

**Affiliations:** ^1^ Department of Medicine, Hazm Mebaireek General Hospital, Hamad Medical Corporation, Doha, Qatar; ^2^ College of Medicine, Qatar University, Doha, Qatar, qu.edu.qa; ^3^ College of Medicine, Weill Cornell Medicine–Qatar, Doha, Qatar, medcol.mw; ^4^ Medical Education Department, Hamad Medical Corporation, Doha, Qatar, hamad.qa

**Keywords:** biphasic hormonal response, central diabetes insipidus (CDI), hemorrhagic pituitary adenoma, pituitary apoplexy, sterile meningitis, syndrome of inappropriate antidiuretic hormone secretion (SIADH)

## Abstract

Pituitary macroadenomas are prevalent benign tumors that can present with insidious symptoms, leading to delayed diagnoses. We report the case of a 29‐year‐old South Asian male who presented with acute neurological symptoms including severe headache, confusion, and vomiting, initially diagnosed as meningitis with syndrome of inappropriate antidiuretic hormone secretion (SIADH). Despite initial improvement, the patient developed central diabetes insipidus (CDI) and was found to have physical features consistent with acromegaly. Magnetic resonance imaging (MRI) revealed a large sellar/suprasellar lesion, and hormonal profile confirmed elevated insulin‐like growth factor 1 (IGF‐1) with low levels of prolactin, luteinizing hormone (LH), follicle‐stimulating hormone (FSH), and testosterone, consistent with pituitary apoplexy. The patient underwent successful transnasal transsphenoidal resection of a hemorrhagic macroadenoma. This case highlights the importance of considering pituitary apoplexy in the differential diagnosis of acute neurological symptoms and its potential for atypical complications such as SIADH followed by CDI. Timely diagnosis and appropriate management are crucial to mitigate risks and improve outcomes.

## 1. Introduction

Pituitary macroadenomas are a significant clinical concern due to their prevalence and potential for serious complications. These benign tumors, arising from the pituitary gland, have an estimated prevalence of 15%–20%, with variations noted between autopsy and imaging studies [1]. Defined as tumors exceeding 10 mm, pituitary macroadenomas have an incidence of approximately 4 per 100,000 persons per year, with prevalence increasing with age [2, 3]. They are classified as functioning adenomas, which secrete hormones and often result in clinical syndromes, and nonfunctioning adenomas. Functioning adenomas carry an increased risk of morbidity and mortality due to hormonal excess, concurrent hypopituitarism, and mass effects [4].

The clinical presentation of pituitary apoplexy can include typical symptoms such as acute severe headache, visual disturbances, and ophthalmoplegia [5, 6], as well as atypical presentations that mimic other conditions, including meningitis‐like symptoms with fever and meningismus, or isolated endocrine disturbances such as SIADH and subsequent CDI [7, 8]. The clinical presentation of pituitary macroadenomas can be insidious, often leading to oversight. Symptoms of pituitary apoplexy may include headache, fever, visual disturbance, and endocrine dysfunction, which can mimic conditions like meningitis [5, 6]. In particular, the acute onset of severe headache, fever, confusion, and hyponatremia, as seen in our case, can lead to an initial presumptive diagnosis of meningitis with syndrome of inappropriate antidiuretic hormone secretion (SIADH) [7].

Timely diagnosis is crucial as delayed diagnosis can result in significant morbidity. Pituitary apoplexy, characterized by sudden hemorrhage or infarction of the adenoma, can lead to acute neurological deficits, including visual loss and hypopituitarism [8]. The risk is particularly pronounced in larger macroadenomas, which may present with acute symptoms requiring immediate intervention [9]. The clinical scenario of a hemorrhagic pituitary macroadenoma presenting as meningitis with subsequent development of SIADH and central diabetes insipidus (CDI) is rare and intricate.

There are reports of pituitary macroadenomas presenting with acute neurological symptoms [10] and hormonal imbalances [11]. However, the combination of meningitis‐like features and SIADH leading to CDI is particularly rare. A study highlighted that patients with elevated serum prolactin levels at presentation had better postsurgery outcomes in terms of recovery of pituitary endocrine function than those with low levels, emphasizing the importance of biochemical markers [12]. Furthermore, the implications of delayed treatment can be profound, leading to irreversible endocrine deficits and diminished quality of life [5, 7].

## 2. Case Presentation

A 29‐year‐old South Asian male with no significant medical history was brought to the emergency department with a two‐day history of severe headache, confusion, and vomiting. On examination, his vital signs showed a temperature of 39 °C, heart rate of 116 bpm, blood pressure of 117/64 mmHg, and SpO2 of 97% on room air. The patient showed positive meningeal signs, but no visual deficits were observed.

**Figure 1:**
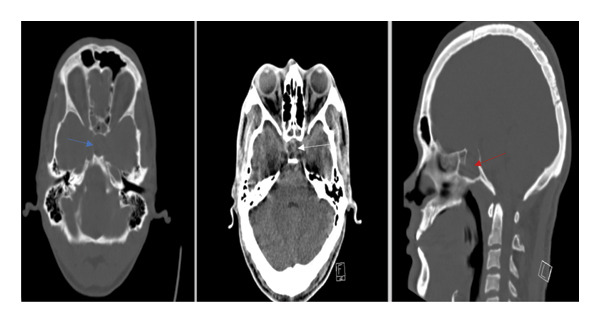
CT scan on admission. Selective nonenhanced CT scan images of the sellar region (axial bone window, axial brain window, and sagittal bone window) show a wide sella with thinning of the sella turcica bone (blue arrow). The sellar fossa is depressed (red arrow) and filled with a heterogeneous lesion without calcifications (white arrow).

Initial laboratory tests revealed severe hyponatremia, and the patient was admitted to a high‐dependency unit (HDU) with a presumptive diagnosis of meningitis and SIADH (Table 1). A noncontrast computed tomography (CT) scan of the head was ordered to rule out space‐occupying lesions or intracranial hemorrhage (Figure 1), and cerebrospinal fluid (CSF) analysis was subsequently performed. As the CT scan was ordered from the ED, no special comment about the sellar area was given in the report. CSF analysis revealed mild total pleocytosis with neutrophilic predominance and a slightly elevated red blood cell count (Table 2). Although euvolemic, given symptomatic hyponatremia, he was started on intravenous hypertonic saline for 2 days with a target correction of 10 mmol of sodium per day. Intravenous fluid was stopped once the serum sodium level was 133 mmol/L. Further laboratory results for hyponatremia at admission were consistent with SIADH, showing low serum osmolality (< 275 mOsm/kg), high urine osmolality (> 100 mOsm/kg), and high urine sodium (> 30 mmol/L) (Table 3). The viral panel and tuberculosis polymerase chain reaction (TB PCR) of the CSF returned negative on the second day. As bacterial cultures from the CSF were pending, the patient continued to receive intravenous ceftriaxone and dexamethasone.

**Table 1:** Complete blood count on admission.

Complete blood count	Patient’s result	Reference ranges
White blood cells	3.2	4.0–10.0 × 10^3^/µL
Red blood cells	5.7	4.5–5.5 × 10^6/µL
Hemoglobin	14.3	13.0–17.0 gm/dL
Hematocrit	39.5	40%–50%
Platelets	158	150–410 × 10^3/µL
Clinical chemistry on admission		
Urea	9.1	2.5–7.8 mmol/L
Creatinine	113	62–106 μmol/L
Sodium	112	133–146 mmol/L
Potassium	4.2	3.5–5.3 mmol/L
Chloride	81	95–108 mmol/L
Bicarbonate	16	22–29 mmol/L
CRP	33.5	0–5 mg/L
Clinical chemistry on 3rd day after admission		
Urea	4.6	2.5–7.8 mmol/L
Creatinine	76	62–106 μmol/L
Sodium	146	133–146 mmol/L
Potassium	4.8	3.5–5.3 mmol/L
Chloride	108	95–108 mmol/L
Bicarbonate	25	22–29 mmol/L

**Table 2:** Cerebrospinal fluid analysis.

Lab parameters	Patient’s result	Reference range
Total nucleated cells in CSF	34	0–5/µL
RBC CSF	12	0–2/µL
Neutrophils CSF %	10	0%–6%
Lymphocytes CSF %	63	40%–80%
Monocyte CSF %	27	15%–45%
CSF glucose	3.18	2.22–3.89 mmol/L
CSF protein	0.83	0.15–0.45 mmol/L
CSF lactic acid	1.9	1.1–2.4 mmol/L

**Table 3:** SIADH and central diabetes insipidus workup.

Lab results taken at time of admission		
Serum osmolality	255	275–295 mmol/kg
Urine osmolality	136	150–1150 mmol/kg
Urine sodium	39	
Lab results on 3rd day after admission		
Serum osmolality	295	275–295 mmol/kg
Urine osmolality	198	150–1150 mmol/kg

On the third day, the patient’s condition improved markedly, with complete resolution of headache and confusion; he was transferred to the general ward. Bacterial cultures of CSF showed no growth. However, he then began experiencing polyuria with an output of approximately 8–10 L versus an intake of 3–4 L. Repeated tests of serum and urine osmolality were consistent with CDI (Tables 1 and 3). Upon further examination, the patient was noted to have physical features suggestive of acromegaly, including large and puffy hands, a prominent nose and chin, and widely spaced teeth. He denied these features were new and reported they had been present for years. Visual field tests revealed no deficits. Thyroid function tests, ordered as part of the hyponatremia workup, indicated central hypothyroidism. Given the constellation of symptoms, a full pituitary hormonal profile was obtained, along with magnetic resonance imaging (MRI) of the pituitary gland.

**Figure 2:**
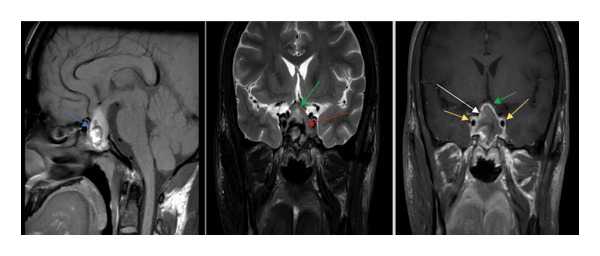
MRI pituitary with contrast (preoperative). Selective images from pituitary MRI scan (sagittal T1WI, coronal T2WI, and postgadolinium coronal image) show a large heterogeneous sellar and suprasellar lesion. It appears heterogeneously hyperintense on T1WI (blue arrow), corresponding to iso‐ to slightly hyperintense on T2WI, with internal focal dark or hypointense signal (red arrow), and shows mild enhancement with more obvious peripheral enhancement (white arrow). The lesion depresses the sphenoid sinus, laterally displaces the carotid arteries bilaterally (yellow arrow), and extends superiorly, elevating the optic chiasm (green arrow).

MRI revealed a large sellar/suprasellar 8‐shaped mass lesion measuring approximately 19 × 29 × 30 mm in the anteroposterior (AP), transverse (TR), and craniocaudal (CC) dimensions, respectively. It had mixed signal intensities on T1‐weighted imaging (T1WI) and T2‐weighted imaging (T2WI). It exhibited an irregular rim postcontrast enhancement. The lesion occupied the enlarged sella turcica, obliterated the suprasellar cistern, elevated the optic chiasm, and slightly indented the hypothalamus/third ventricle. It extended to the cavernous sinuses, more on the left side, and sagged inferiorly into the sphenoid sinus; a radiological diagnosis of hemorrhagic pituitary macro adenoma was made (Figure 2). The hormonal profile confirmed elevated insulin‐like growth factor 1 (IGF‐1) with low levels of prolactin, luteinizing hormone (LH), follicle‐stimulating hormone (FSH), cortisol, and testosterone, consistent with pituitary apoplexy (Table 4). The endocrinology team confirmed the clinical diagnosis of pituitary apoplexy with CDI and possible panhypopituitarism. They recommended starting intranasal desmopressin and levothyroxine and shifting dexamethasone to hydrocortisone.

**Table 4:** Hormonal profile at presentation and postsurgical resection of hemorrhagic adenoma.

Lab parameter	Initial hormonal profile	1 month on follow‐up	Reference range
ACTH	19	14.9	7.2–63.3 pg/mL
Cortisol	12.2 AM	441 AM	AM 138–689 nmol/L
Follicle‐stimulating hormone	1.4	2.2	1.5–12.4 IU/L
Luteinizing hormone	1.2	3.1	1.7–8.6 IU/L
Testosterone	< 0.09	L 1.65	10–37 nmol/L
TSH	0.20	1.23	0.3–4.20 mIU/L
Free T3	2.0		3.7–6.4 pmol/L
Free T4	9.9	14.9	11–43 pmol/L
Prolactin	6	L 22	85–323 mIU/L
Insulin‐like growth factor (IGF‐1)	796	L 119	120–295 μg/L
Human growth hormone (HGH)	3.71	0.09	Ug/L

Abbreviations: RBC, red blood cell; ACTH, adrenocorticotrophic hormone; T3, triiodothyronine; T4, thyroxine.

Interestingly, the patient remained hemodynamically stable, likely due to the ongoing administration of dexamethasone (8 mg three times daily), continued for the presumptive diagnosis of meningitis. Dexamethasone is preferred as it does not cross‐react with cortisol assays.

## 3. Outcome and Follow‐Up

**Figure 3:**
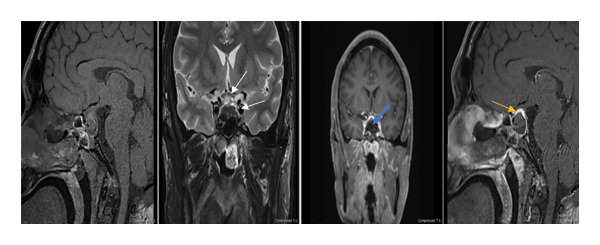
MRI pituitary with contrast (postoperative). Postoperative selective MRI images of the pituitary region (sagittal T1WI, coronal T2WI, and post‐IV contrast coronal and sagittal images) show an empty sellar and suprasellar fossa with reduced compressive effects on the chiasm and carotids (white arrow), peripheral enhancement (yellow arrow), and no soft tissue component (blue arrow).

The patient was transferred to neurosurgery and underwent successful endoscopic transnasal transsphenoidal resection of the hemorrhagic pituitary macroadenoma (Figure 3). Postoperatively, hydrocortisone was gradually tapered, desmopressin was stopped after significant improvement in urine output, and levothyroxine was continued until discharge. Histopathology revealed hemorrhagic necrosis of the infarcted pituitary tumor, consistent with apoplexy.

One month postresection, the patient’s hormonal profile showed significant improvement, with decreased IGF‐1 and growth hormone levels (Table 4). The patient attended outpatient endocrinology follow‐up 1 week later with no new symptoms, with a plan to continue levothyroxine and regular follow‐up.

## 4. Discussion

In approximately 95% of the patients with acromegaly, the cause of growth hormone hypersecretion is a somatotroph pituitary adenoma [13]. However, diagnosis is often delayed by an average of 10–12 years due to the subtle progression of physical findings that patients may not notice [14]. As in our case, when asked about his physical findings, the patient failed to recognize the changes. Therefore, even if the patient denies changes, clinicians should maintain a high index of suspicion for both functional and nonfunctioning pituitary neuroendocrine tumors (Pit‐NETs) to avoid overlooking pituitary dysfunction. Physicians should be vigilant for red flags such as acute visual changes, severe headaches, altered mental status, or hormonal disturbances. Regular check‐ups can also prevent such conditions from being overlooked. A thorough review of the patient’s medical history, tailored to clinical stability, is essential for accurate assessment and timely intervention.

Pituitary apoplexy is a rare but potentially fatal complication of pituitary adenomas, resulting from hemorrhage, infarction, or hemorrhagic infarction within the tumor. It occurs in 2%–12% of patients with pituitary adenoma, with an incidence around 0.17 episodes per 100,000 patients per year [15]. The prevalence among prolactin, growth hormone, and ACTH‐secreting tumors is 6–10, 5.3–6, and 3.9 cases per 100,000 inhabitants, respectively. The prevalence among nonfunctioning pituitary adenomas turning to apoplexy ranges from 7 to 10 cases per 100,000 inhabitants [16]. Thus, growth hormone‐secreting pituitary adenoma is the second most common functioning tumor to convert into pituitary apoplexy.

The clinical presentation of pituitary apoplexy varies from acute severe headache with vomiting to visual disturbances, cranial nerve palsies, loss of consciousness, and meningismus. Although CSF findings vary, they can include elevations in white blood cells, red blood cells, and protein [17]. Our patient’s CSF showed neutrophil‐predominant pleocytosis. These findings can be attributed to the spillage of blood and necrotic material from the hemorrhagic pituitary gland into the subarachnoid space, causing chemical meningitis [18]. This spillage caused our patient’s symptoms of fever, vomiting, and meningismus.

The CT scan in our patient was ordered from the ED to rule out acute hemorrhage. Reviewing the CT films with focus on the sellar area revealed widening of the sella and thinning of the sella turcica bone. Although suggestive of pituitary pathology, these findings are neither sensitive nor specific for ruling out pituitary apoplexy, as studies show CT detects only 21% of the cases, whereas 90% are visualized with MRI [19, 20]. MRI is superior for assessing pituitary and parasellar structures compared with CT [21]. Additionally, MRI is more specific for ruling out pituitary apoplexy and detecting hemorrhagic elements [22].

Hyponatremia due to SIADH is a well‐known complication of neurosurgery, brain trauma, and meningitis [23, 24]. A triphasic response following pituitary surgery characterized by acute hypernatremia, transient hyponatremia (approximately 3–11 postoperative days), and subsequent CDI is documented [26, 27]. We suspect the initial SIADH/hyponatremia in our patient occurred through a mechanism similar to pituitary surgery or brain trauma, involving an acute outburst of ADH from damaged posterior pituitary cells [24, 25]. This damage can subsequently lead to CDI due to depletion of stored ADH, which may resolve in weeks or become permanent [24]. Hyponatremia is common in hospitalized patients, and pituitary apoplexy is often overlooked. The absence of visual deficits, denial of physical findings, and initial unremarkable head CT led to delayed MRI and final diagnosis in our case.

Pituitary apoplexy, although a rare differential diagnosis in patients with acute headache and confusion, should be considered in patients with symptoms or findings suggestive of a pituitary adenoma and must be ruled out promptly to decrease associated morbidity and mortality.

## 5. Conclusion

Although benign, pituitary macroadenomas often present with insidious or atypical symptoms, leading to a delayed diagnosis. This case highlights the diagnostic challenges of pituitary apoplexy, particularly when it mimics conditions, such as meningitis, or presents with a biphasic hormonal response (SIADH followed by CDI). The patient’s initial presentation with acute neurological symptoms and hyponatremia, initially attributed to meningitis and SIADH, underscores the importance of considering pituitary apoplexy in patients with severe headaches, confusion, and hormonal disturbances, even without classic visual deficits.

Clinicians should remain vigilant for both typical (e.g., visual deficits and hormonal imbalances) and atypical (e.g., meningitis‐like symptoms and biphasic hormonal response) presentations. Physical features such as acromegaly can provide critical diagnostic clues. Early MRI is essential because CT scans may miss early signs of pituitary apoplexy. Timely surgical intervention, as demonstrated in this case, is crucial for preventing long‐term morbidities.

This case reinforces the need to include pituitary apoplexy in the differential diagnosis of acute neurological and endocrine disorders. By maintaining a high index of suspicion and utilizing appropriate diagnostic tools, clinicians can improve the outcomes of patients with this rare but potentially life‐threatening condition.

## 6. Key Clinical Message

Pituitary apoplexy, although rare, can mimic meningitis and present with a biphasic hormonal response of SIADH initially followed by CDI. Early MRI and consideration of pituitary apoplexy in the differential diagnosis of acute neurological symptoms are crucial for timely intervention and optimal patient outcomes.

## Author Contributions

Abdulwahab Muhammad Qasem: Data collection, literature review, and manuscript writing. (draft and final editing). Bassem Al Hariri: supervision, writing–original draft, and writing–review and editing. Muhammad Faizan: writing–original draft and writing–review and editing. Reynaldo Balintona Jr: writing–original draft and writing–review and editing. Mohammed Omer Elbadawi Elhassan: writing–original draft and writing–review and editing. Suhib Salameh: writing–original draft and writing–review and editing. Imran Hussain Mohammad: writing–original draft and writing–review and editing. Abdulwahab Muhammad Qasem: writing–original draft and writing–review and editing.

## Funding

This case report was not funded.

## Disclosure

All authors read and approved the final manuscript.

## Ethics Statement

The patient provided oral and signed written consent to use his clinical materials in this study. The study was conducted by the principles of the institutional ethical standards and the national research committee.

## Consent

Written informed consent was obtained from the patient for publication of this case report and accompanying images with the journal’s patient consent policy.

## Conflicts of Interest

The authors declare no conflicts of interest.

## Data Availability

The data that support the findings of this study are available from the corresponding author upon reasonable request.
